# Notes and News

**Published:** 1902-04-19

**Authors:** 


### Notes and News.

The Queen continues to take an interest in the Finsen Light treatment for lupus, and during her visit to Copenhagen has visited Professor Finsen's Light Institute. She has also been to see several hospitals.

The Essex County Council have received, in reply to their protest against the use of the small-pox ships, a reply in the House of Commons from Mr. Long, that the Government cannot interfere with the discretion of the authorities.

The late Miss Lydia Hale, of Blackheath, has bequeathed £200 each to St. Thomas's Hospital, the Charing Cross Hospital, and the Central London Ophthalmic Hospital, and £100 each to the Central London Throat and Ear Hospital and to the Blackheath and Charlton Cottage Hospital.

The late Mr. Irvine, of Granton, Edinburgh, has left instructions to his trustees to set aside a portion of his shares in a business with which he was connected, and to allow the interest to accumulate until a capital of £25,000 to £30,000 is secured. This sum is then to be handed over to the University of Edinburgh to found a Chair of Bacteriology.

The managers of the Central Sick Asylums, Cleveland Street and Hendon, at their meeting, Monday, April 7th, 1902, decided to establish a department for the X-ray treatment of lupus. We are informed that there are about 25 cases in the metropolitan area, and, with the permission of the Local Government Board, these can be sent to Hendon at a certain charge.

We regret to learn that Dr. MacAlister has resigned the post of physician to Addenbrooke's Hospital, Cambridge. He has rendered most valuable services there, and he will be much missed. We are glad that he will not give up his connection with the hospital. He will continue to lecture as Linacre Reader of Physic, and remain on the Advisory Council. The position of consulting physician has been offered to Dr. MacAlister. He has acted as physician to Addenbrooke's Hospital for 20 years, and retires from its active service for reasons of health.

Small-pox in London again shows a decided decrease.

The late Mr. G. King, of Eastbourne, has bequeathed £30,000 to Guy's Hospital. The bequest is subject to two life interests.

Sixteen medical officers who were sent out for plague duty to India have received commissions in the Indian Medical Service.

It is stated that bills for medical attendance on the late President McKinley, amounting to about £20,000, will be presented to Congress for payment.

A cottage hospital is to be erected at Nice to honour the memory of Queen Victoria. Many donations have already been made for the purpose.

The Department of Health of the State of New York, announce that they will supply diphtheria antitoxin free to all persons or institutions unable to pay for it.

A report from India, commenting on the satisfactory results from inoculation for the plague, points out that an indirect good also is shown in the increased readiness of the inoculated who escape the disease to adopt sanitary measures as well.

If any proof were necessary to show that the race is becoming less robust in its attitude towards pain it would not be necessary to go further than the columns of our newspapers. It is by no means uncommon nowadays to find there reports of cases of suicide through fear. Quite recently a man was reported as committing suicide to escape vaccination, and a woman cut her throat rather than undergo an operation.

The Secretary of State for War has decided that although the Volunteer Medical Staff Corps, officers and men, have been affiliated to the Royal Army Medical Corps under the title of the Royal Army Medical Corps (Volunteers), and the compound title of the officers abolished it is not considered desirable to interfere with the position of medical officers belonging to regiments or corps, their *status* being quite different from that of officers of the Royal Army Medical Corps (Volunteers).

A combatant officer has written to the *Times* drawing attention to the relaxation of discipline which has permeated our army in South Africa, and to the extent to which this has favoured the spread of disease—especially enteric fever. He says that while the medical authorities made efforts to enforce sanitation, these were often frustrated by apathy or by want of control over their men on the part of commanding officers. The difference between different regiments in this respect was often striking, and he maintains, very properly, as we think, that commanding officers incur a grave responsibility if they fold their hands and say that the prevention of enteric and the sanitation of camps rests entirely with the medical department.

